# Immunotherapy trials in Parkinson’s disease: challenges

**DOI:** 10.1186/s12967-023-04012-x

**Published:** 2023-03-06

**Authors:** Bin Xiao, Eng-King Tan

**Affiliations:** 1 Department of Neurology, National Neuroscience Institute, Singapore, Singapore; 2 Neuroscience and Behavioral Disorders Program, Duke-NUS Medical School, Singapore, Singapore

**Keywords:** Clinical trial, α-synuclein, Antibody, Immunotherapy

## Main text

Parkinson’s disease (PD) is a common neurodegenerative disorder, and its incidence increases from age 60 to over 90 years, posing a serious social and economic burden on aging populations globally. Point missense mutations and gene duplication or triplication of α-synuclein gene are linked to familial PD. Genome-wide association study-linked variants of α-synuclein also contribute to the risk of sporadic PD. Exogenous expression of wild-type or mutant α-synuclein recapitulates PD-like features in various animal models. The non-amyloid component domain of α-synuclein protein comprises eight β-strands and predisposes the protein to aggregation to form fibrils. Fibrillar aggregates of α-synuclein are found in Lewy bodies (LBs), a pathological hallmark of PD. Hence targeting α-synuclein has been one of the major focuses of PD therapeutics (reviewed in [1, 2]). Both passive and active immunotherapy trials against α-synuclein have created considerable excitement and hope [3].

Lang et al. reported the results of the SPARK trial that early-stage PD patients received Cinpanemab (a monoclonal antibody that preferentially binds to aggregated α-synuclein at the N-terminal of the protein to promote clearance of extracellular α-synuclein, mitigating pathological consequences [4]) experienced similar progression rate of symptoms compared with placebo up to 72 weeks [5]. In parallel, Pagano et al. reported the findings from the PASADENA study where Prasinezumab (a monoclonal antibody directed against aggregated α-synuclein at the C-terminal of the protein, which may attenuate neurodegeneration by blocking the spread of pathogenic α-synuclein between neurons [6]) failed to slow down the PD disease progression over 52 weeks [7]. Consistently, dopamine transporter imaging with single-photon-emission computed tomography (SPECT) showed little differences between the control group and treated group in both the trials [5, 7].

These clinical trials were preceded by robust findings using Cinpanemab and Prasinezumab in preclinical rodent models, where significant improvement of PD-like phenotypes has been observed [6, 8]. Phase I clinical trials in healthy subjects and patients with PD found Cinpanemab/α-synuclein complex formation in plasma [9] and dose-dependent reduction of serum α-synuclein levels by Prasinezumab [10]. What could have accounted for these divergent outcomes? The susceptibility and compensatory mechanisms pertaining to the pathogenesis of PD in animal models differ from those in humans. In addition, both genetic and toxin-induced animal models involve either artificial overexpression of the genes or chemical induction over a short period of time, events that may not reflect the actual situations in the human brain. Patient-derived induced pluripotent stem cells (iPSCs) may be differentiated into neurons/midbrain organoids with pathological features, including LB-like inclusions, thus better recapitulating the pathological changes [11]. These human models together with nonhuman primate models can be tested in future preclinical trials.

Placebo-related improvements have been observed in PD clinical trials and it may not diminish at 6 months of follow-up [12]. In addition, perceptions of medication cost were capable of altering the placebo response in a PD clinical study [13]. In the studies by Lang et al. and Pagano et al., it is unclear if perceptions of being involved in a state-of-art monoclonal antibody clinical trial have created a more profound placebo effect. A longer follow-up period may help to rule out this possibility.

Both clinical trials recruited the early-stage PD patients, as the downstream pathological cascades in the late-stage PD may already be irreversible even if the inceptive pathogenic drivers are arrested. However, owing to the mechanisms of neural compensation, there is usually 50–60% of DA neuron loss in the substantia nigra of the diagnosed PD patients [14]. The vicious cascades may have been tiggered in the surviving neurons, and therefore therapeutics may be doomed to fail. Future clinical trials may include subjects who are at prodromal stage of PD which could be selected out using criteria developed by the International Parkinson and Movement Disorder Society (MDS), including clinical features, such as rapid eye movement sleep behavior disorder (RBD) and olfactory dysfunction [15].

Over two centuries of research has reached the consensus on the PD pathogenesis that various factors/pathways trigger the pathological events that eventually lead to the disease [1]. If α-synuclein aggregation is not the critical downstream process, targeting it with α-synuclein monoclonal antibodies may ultimately not be effective for the general PD population. Instead, only a sub-group of patients associated with “high α-synuclein load” may benefit from α-synuclein monoclonal antibodies and should be pre-selected for the clinical trials of such therapeutics, particularly carriers of α-synuclein risk variants/mutations and those with high polygenic risk scores in the SNCA gene. Moreover, we will need better quantitative measures to evaluate different α-synuclein forms. Detection of α-synuclein oligomerization/aggregation in cerebrospinal fluid using protein misfolding cyclic amplification (PMCA) or real-time quaking-induced conversion (RT-QuIC) could be potential options. To address the heterogeneity of PD pathogenesis, a cocktail therapy that targets the core upstream pathogenic pathways together with α-synuclein immunotherapy may be a consideration for future trials.

In targeted immunotherapy, perhaps clinical assessment and imaging dopamine integrity alone may not be enough. Motor assessment scales (such as UPDRS) may not be an optimal clinical assessment due to its intrinsic weakness, including inter-rater consistency [16]. Functional imaging (such as SPECT) may reflect the nigrostriatal degeneration, but not improved pathological changes in the neurons which may experience better outcome in the long run. Thus, assessment of the burden of α-synuclein aggregation with the aid of iPSC-derived neurons and α-synuclein neuroimaging probes may be able to capture the difference in the clinical outcomes.

In summary, while the SPARK [5] and PASADENA [7] studies did not demonstrate efficacy of α-synuclein monoclonal antibodies in early PD, the results highlight the need to review potential limitations of preclinical studies and clinical trials from experimental models to endpoint assessments. The introduction of emerging technologies may help address the challenges in the α-synuclein-based immunotherapy. Despite the disappointing results, it may be premature to abandon exploring the potential of such therapeutic approaches.

## Data Availability

Not applicable.

## Abbreviations

PD: Parkinson’s disease
LBs: Lewy bodies
SPECT: Single-photon-emission computed tomography
iPSCs: Patient-derived induced pluripotent stem cells
MDS: International parkinson and movement disorder society
RBD: Rapid eye movement sleep behaviour disorder
PMCA: Protein misfolding cyclic amplification
RT-QuIC: Real-time quaking-induced conversion
